# Mechanical Properties and Bioactivity of Polyetheretherketone/Hydroxyapatite/Carbon Fiber Composite Prepared by the Mechanofusion Process

**DOI:** 10.3390/polym13121978

**Published:** 2021-06-16

**Authors:** In Sung Jeon, Moon Hyun Lee, Han-Hyeong Choi, Sangwoon Lee, Joon Woo Chon, Dong June Chung, Jong Hyuk Park, Jae Young Jho

**Affiliations:** 1 School of Chemical and Biological Engineering, Seoul National University, Seoul 08826, Korea; ajeon88@gmail.com (I.S.J.); tttyyy0403@snu.ac.kr (S.L.); 2 Department of Polymer Science & Engineering, Sungkyunkwan University Suwon, Suwon 16419, Korea; bluehyun93@skku.edu (M.H.L.); chonjoon@skku.edu (J.W.C.); 3 Soft Hybrid Materials Research Center, Korea Institute of Science and Technology, Seoul 02792, Korea; onebro@snu.ac.kr (H.-H.C.); hyuk0326@kist.re.kr (J.H.P.)

**Keywords:** PEEK/HA/CF composite, mechanical property, suspension blending, mechanofusion, in vitro biosafety

## Abstract

The main obstacles in the melt-processing of hydroxyapatite (HA) and carbon fiber (CF) reinforced polyetheretherketone (PEEK) composite are the high melting temperature of PEEK, poor dispersion of HA nanofillers, and poor processability due to high filler content. In this study, we prepared PEEK/HA/CF ternary composite using two different non-melt blending methods; suspension blending (SUS) in ethanol and mechanofusion process (MF) in dry condition. We compared the mechanical properties and bioactivity of the composite in a spinal cage application in the orthopedic field. Results showed that the PEEK/HA/CF composite made by the MF method exhibited higher flexural and compressive strengths than the composite prepared by the SUS method due to the enhanced dispersibility of HA nanofiller. On the basis of in vitro cell compatibility and cell attachment tests, PEEK/HA/CF composite by mechanofusion process showed an improvement in in vitro bioactivity and osteo-compatibility.

## 1. Introduction

In spinal cage applications, metals such as titanium (Ti) and stainless steel have been widely used for metallic implants due to their excellent corrosion resistance, biocompatibility, mechanical strength, and friction resistance. For example, Ti-6Al-4V, one of the titanium alloys, exhibits outstanding biocompatible and corrosion resistance [1]. However, mismatches in the Young’s modulus, magnetic image interference, and release of ions are major issues [2]. Glass-ceramics such as Apatite-Wollastonite (A-W) are also used in the spinal cage application due to their good biocompatibility, low cost, and ware resistance. However, the use of A-W glass-ceramic is often limited because of its brittleness and poor handling properties [3,4].

A large number of polymers such as polyethylene (PE), polyethylene terephthalate (PET), polysulfone (PS), poly(lactic acid) (PLA), and poly(glycolic acid) (PGA) have been used in specific biomedical applications [5]. Nevertheless, these polymers are not suitable for use as a spinal cage application due to their low mechanical strength and modulus. Thereby, polyetheretherketone (PEEK) has been a primary candidate to replace metallic implants because of its good chemical resistance, biocompatibility, mechanical strength, and MRI compatibility [6,7,8]. While metallic implants often result in bone resorption and osteonecrosis due to a stress-shielding effect [9,10] from a much higher Young’s modulus (102–110 GPa) than that of the natural human bone (~14 GPa), PEEK has a similar Young’s modulus (3–4 GPa) to human bone and mitigate these issues [11,12,13].

Nonetheless, PEEK needs some improvements to be used in a spinal cage application. Since the mechanical strength and elastic modulus of PEEK itself is in the low range of human cortical bone, PEEK should be further reinforced to match up with the mechanical properties of cortical bone. Carbon fiber (CF) is commonly used as a reinforcement in PEEK composite due to its excellent mechanical properties, biocompatibility, wearability, non-toxicity, and low cost [14,15]. Carbon fiber-reinforced PEEK (CFRPEEK) is currently used in the orthopedic field for various applications such as spinal cage, joint replacement, and plates [16]. In terms of mechanical properties, the higher the CF content in the composite, the better it is. However, the maximum content of CF in the composite should be adjusted due to the hydrophobicity, which can weaken the cell attachment, spreading, and proliferation [17]. Sandler et al. [13] reported that the tensile stiffness and strength of a PEEK composite can be enhanced by the addition of carbon nanofibers (CNF) due to increased surface area and energy of filler. In addition to CF, several studies in the literature have investigated the effect of various nanoparticles on the mechanical properties of PEEK. For example, Hwang et al. [18] improved the bending elastic modulus and damping properties of PEEK with the addition of graphene oxide (GO) and carbon nanotube (CNT). The friction and wear properties of PEEK also could be enhanced by the addition of GO [19]. However, in some cases, the nanoparticle-like graphene nanoplatelet can form an aggregation that can reduce the mechanical strength of PEEK composites [20].

Another factor that must be improved is bioactivity. Even though PEEK is a biocompatible polymer, it does not osseointegrate in vivo and does not provoke interactions with bone tissue [21]. In order to improve the bioactivity of PEEK, one of the bioactive ceramics that has been commonly used is hydroxyapatite (HA), a bioactive calcium phosphate that has a similar chemical composition to human bone. HA is known to promote osteoblast adhesion and cell proliferation by its osteoconductive abilities [22,23,24]. However, nano-sized HA filler within the PEEK/HA composite can be easily agglomerated with increasing content due to its good hydrophilicity and high surface area [25,26]. In addition, aggregation of the HA filler leads to a severe reduction in mechanical strengths and modulus.

Many studies have reported different methods to improve the dispersibility of the HA nanoparticles in the polymer matrix. Mathieu et al. [27] produced a homogeneous dispersion of the HA in the PLA composite by the melt-extrusion method, but pointed out the risk of polymer degradation. An internal mixer (Haake) is another way to disperse nanoparticles in the composite by mechanical mixing force [28]. The dispersibility of HA also can be improved by modifying HA with the silane coupling agent. Ma et al. [29] reported that the surface-modified HA enhanced the tensile strength of the PEEK/HA composite by improving the dispersibility of HA and the interfacial adhesion between the HA and PEEK matrix. Wang et al. [2] developed a PEEK composite with nanofluorohydroxyapatite (FHA). Their results show FHA filler not only increased the elastic modulus and tensile strength similar to those of human cortical bone but also enhanced bioactivity, osseointegration, and bone–implant contact in vivo.

Some recent studies have been conducted involving HA and CF fillers incorporated in the PEEK matrix, as PEEK/HA/CF ternary composite in order to improve both the mechanical properties and bioactivity at the same time [17,30]. Even though PEEK/HA/CF ternary composite was enhanced in both mechanical properties and biological performances compared to pure PEEK matrix, dispersibility of fillers in ternary composite was a major issue that has to be improved. In other words, the content of CF in ternary composite should be adjusted at the level of mechanical properties similar to bones, whereas the content of HA should be limited in a way that mechanical properties are not severely deteriorated due to aggregation.

A common method for PEEK composite fabrication in industrial fields is the melt-processing method using an extruder equipped with a high-temperature heater. However, such a method often cannot provide sufficient dispersibility for nano- and micro-sized agglomerated fillers in high contents.

Ultrasonication followed by suspension blending in ethanol is one of the methods that can be done easily in the lab. The sonication treatment is a form of vibration that generates cavitation or bubbles and provides high intensity of ultrasound energy to the filler [31]. By applying sonication and suspension blending in ethanol, the HA aggregates can be disintegrated and uniformly dispersed in the ethanol suspensions without significant deformation or defects [32]. However, PEEK composite fabrication by suspension blending (SUS) is not convenient either in industrial aspects due to low processability, high production cost, and environmental pollution by the solvents used. Given these conditions, mechanofusion (MF), which is a simple and inexpensive high-throughput compounding system, is an adequate approach to enhance both dispersibility of aggregated filler and compatibility between polymer matrix and filler by high shear and compression forces [33,34,35,36]. By MF process, HA particles can be dry-coated onto the surface of PEEK powder, producing a mechanochemical reaction between the host particle, PEEK, and the guest particle, HA [37].

Since the mechanical forces produced by a rotating blade in the chamber break down the fine particle agglomerates, cohesive HA aggregates in PEEK/HA/CF ternary composite can be mechanically dispersed. Moreover, the whole process is cost-effective and environmentally friendly since the MF method can be carried out in dry conditions without any solvents. From the commercial point of view, the manufacturing of PEEK/HA/CF composite at a large scale can be accomplished by using NC-400-P model (Hosokawa Micron), which has a capacity of 10 to 100 kg/hr. To the best of our knowledge, there is no report on the mechanical properties of PEEK/HANF/CF composite prepared by mechanofusion process so far. Hence, this study was conducted to investigate the effect of the non-melt blending process (suspension blending and mechanofusion processing) for PEEK/HA/CF ternary composite on its mechanical properties. We also evaluated in vitro cell compatibility and cell attachment to test the in vitro bioactivity and osteo-compatibility in a spinal cage application.

## 2. Materials and Methods

### 2.1. Materials

PEEK powder (Victrex^®^ 450PF) was purchased from Dict Co. (Seoul, Korea). The commercial HA (Nano HAP04) was purchased from Nanjing Emperor Nano Material Co. Ltd. (Nanjing, China). Carbon fiber (PX 35) was obtained from Zoltek (St. Louis, MI, USA). The detailed properties of materials are shown in Table 1. Human osteoblast (HOB) cells were obtained from Cell Application Inc. (San Diego, CA, USA). Ninety-six-well cell culture plates were purchased from SPL Life Science (Pocheon, Korea). Phosphate-buffered saline (pH 7.4), penicillin-streptomycin, and trypsin-EDTA were purchased from Sigma-Aldrich Chem. Co. (St. Louis, MO, USA). Paraformaldehyde for cell fixing was purchased from Samchun Chem. (Seoul, Korea). Fetal bovine serum (FBS) and Dulbecco’s Modified Eagle Medium with high glucose (DMEM) were purchased from GE Healthcare Life Sciences (Logan, UT, USA). The alkaline phosphatase (ALP) assay kit was purchased from AnaSpec. Co. Inc. (Fremont, CA, USA). All the other chemicals were purchased from Sigma-Aldrich Corporation.

### 2.2. Preparation of PEEK Composite Powders

Two kinds of reinforcements (HA and CF) were blended with the PEEK powder, according to the composition provided in Table 2. To prepare composite powders using SUS method, PEEK powder and fillers were separately dispersed in ethanol, followed by ultrasonication for 60 min. Then, each dispersed powder was mixed with another using a magnetic stirrer for 12 h. The composite suspension was filtered and dried at 110 °C for 24 h.

**Figure 1:**
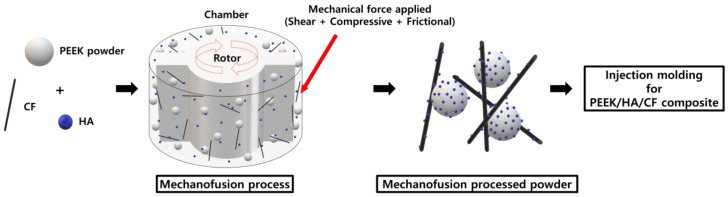
A schematic of mechanofusion process to prepare the PEEK/HA/CF composite.

The equipment for the MF process (Nanocular System, Hosokawa Micron) included a reaction chamber with a rotor that applied strong shear, compressive, and frictional forces to the blended materials [38]. The PEEK, HA, and CF powders were placed in the chamber according to Table 2. A schematic of the mechanofusion process to prepare PEEK/HA/CF composite is shown in Figure 1. Due to the difficulty in the injection process, the maximum amount of the HA and CF fillers was limited to below 40 wt%. To achieve high levels of dispersion through exfoliation of the aggregated HA, the rotor was rotated at 2500 rpm for 1 h. During the process, the chamber was cooled by circulating water to maintain a constant temperature.

### 2.3. Characterization and Measurement of PEEK Composites

The surface morphologies of PEEK and CF were characterized by scanning electron microscopy (SEM; Sigma 300; ZEISS). The surface composition of PEEK and CF was determined using energy dispersive spectrometry (EDS), which was coupled with SEM. The fractured surfaces of PEEK/HA/CF composites after flexural testing were also characterized by SEM. The morphology and dispersion state of the HA in the composites were observed by X-ray micro-computed tomography (micro-CT) (SkyScan 1172, Bruker) [39,40]. The detailed conditions for the micro-CT measurements were as follows. X-ray source voltage and current 42 kV and 240 μA, respectively; monitored sample size ca. 1 × 1 × 1 mm^3^; projection numbers per sample 1800; exposure time per projection 3200 ms. Micro-CT images of the composites were obtained by reconstructing the projections. The size distribution of HA in the composites was investigated using software (CTvox) for three-dimensional modeling.

To evaluate the mechanical properties of PEEK composites, every composite powder made by both SUS and MF methods was prepared using a mini-injection molding machine (Bautek Co., Uijeongbu-si, Korea) at processing temperatures of 390 °C. The pre-set molding temperature was set to 190 °C. The composite powders were molded into 80 × 10 × 4 mm^3^ and 10 × 10 × 4 mm^3^ plates for flexural and compressive tests, respectively. Then, the composite samples were annealed at 220 °C for 4 h to provide a similar degree of crystallinity. Test specimens of 10 × 10 × 4 mm^3^ were also used for the in vitro cell compatibility and cell attachment tests.

The flexural and compressive properties were measured with a universal testing machine (Lloyd LR10K, West Sussex, UK) with a load cell of 10 kN, according to the ISO 178 and ISO 604 standards. The cross-head speeds were 2.0 mm/min and 1.0 mm/min for flexural and compression tests, respectively. The average of five measurements was obtained from seven specimens for each test.

### 2.4. In Vitro Cell Compatibility Test

To evaluate the cell compatibility of the composites, every composite was extracted for 24 h at 36.5 °C with DMEM medium. The extracted composite solution was diluted to various concentrations (1, 5, 10 and 20%). The HOB cells were seeded in each well (1.0 × 10^4^ cells/well) of 96-well cell culture plates (TCPS). An amount of 100 µL of ALP solution containing pNPP (p-nitrophenyl phosphate) was added to each well. Subsequently, the cells were cultivated for 1 h at 36.5 °C in 5% CO_2_ condition and 50 µL of stop solution in ALP assay kit was added into each well. To determine the cell activity, the ultraviolet-visible (UV-Vis) absorbance of the solutions in each well was measured at 405 nm using a Varioskan LUX multimode microplate reader (Thermo Fisher Scientific, Waltham, MA, USA).

### 2.5. Cell Attachment Test

The HOB cell adhesion behavior on the surface of composites was determined by the cell attachment test. The cells were seeded on the surface of composites with identical cell density (2.0 × 10^4^ cells per well) for 24 h of cultivation. And then, the surface of composites was rinsed with PBS solution to remove the unattached cells. To observe the morphology of HOB cells which were attached to the surface of composites, the HOB cells were fixed with 4 wt% paraformaldehyde solution for 30 min. The morphology of HOB cells was observed by Olympus BX51M optical microscope (Olympus, Tokyo, Japan) and JSM-7500F scanning electron microscope (JEOL, Tokyo, Japan).

## 3. Results and Discussion

### 3.1. Morphology of Powders

**Figure 2:**
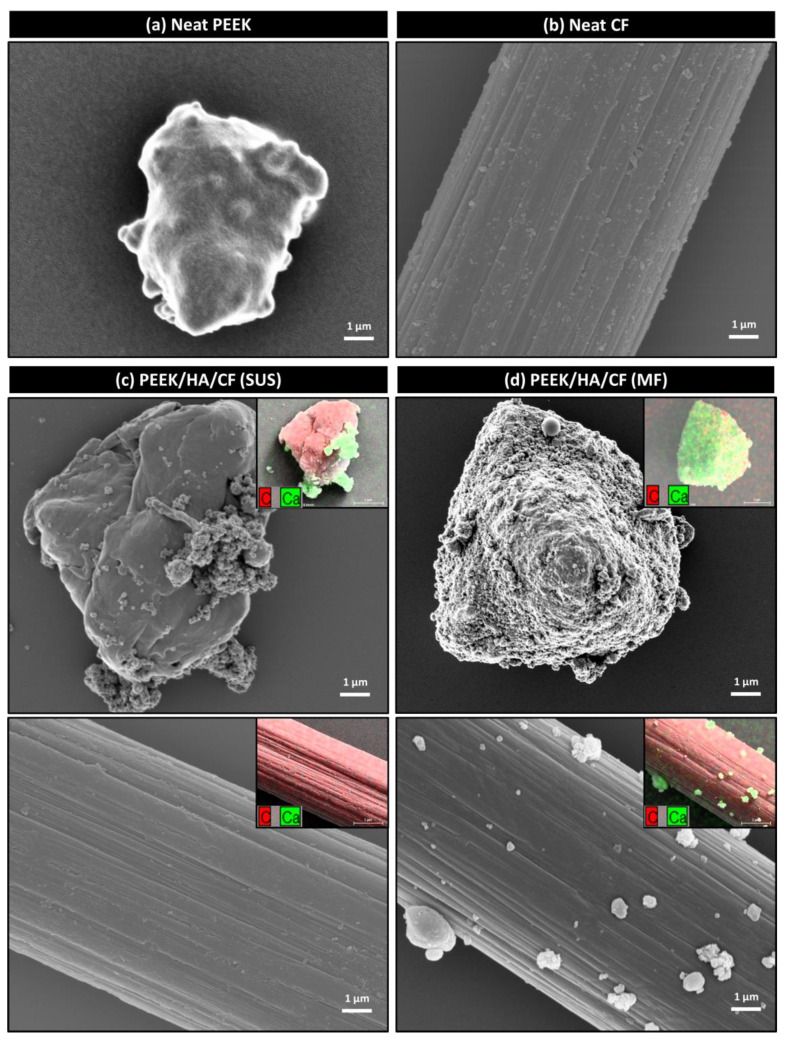
SEM images of (**a**) PEEK and (**b**) CF surfaces; SEM images and corresponding EDS mapping of C and Ca for PEEK/HA/CF composite powder on PEEK and CF surfaces prepared by (**c**) SUS and (**d**) MF methods.

The surface morphologies of PEEK and CF particles in PEEK/HA/CF composite powders prepared by SUS and MF methods are shown in Figure 2. The surfaces of neat PEEK (Figure 2a) and CF (Figure 2b) particles before any treatment exhibited smooth and clean surfaces. As shown in Figure 2c, HA aggregates were adhered to the surface of PEEK particles, but not the surface of CF. On the other hand, the surfaces of both PEEK and CF were covered with HA fillers after MF process (Figure 2d). The attached HA particles were confirmed by EDS mapping of Ca. The MF processing could induce the mechanical interlocking at the interface of different components by applying strong mechanical forces [41,42]. PEEK/HA/CF composite fabricated using HA-covered PEEK and CF was expected to show improved interfacial adhesion between PEEK matrix and fillers. Furthermore, molten PEEK during the injection molding process was expected to penetrate the microgaps formed between HA particles on the CF surface, resulting in improved interfacial strength through mechanical interlocking between components [43].

### 3.2. Mechanical Properties

The results of flexural strength test are shown in Table 3. Composite samples prepared by both SUS and MF method showed an increase in flexural strengths and modulus as the CF content increased. The flexural strengths of C1, C2, and C3 made by MF process were 185, 221, and 254 MPa, respectively. Compared to C1, C2, and C3 samples made by the SUS method, mechanofused samples showed similar values in the flexural strengths within the error range. The applied mechanical forces by MF process were not effective on the micro-sized CF fillers compared to the nano-sized HA fillers that are easily aggregated. Due to the high modulus and stiffness characteristics of the CF filler [44], the flexural moduli for samples prepared by both methods were increased as with increasing CF content. In contrast, the flexural strains at the break decreased with an increasing CF content due to the rigid characteristics of CF filler, resulting in fracture-behavior transition from ductile to brittle.

When the HA filler was added to the composite, both flexural strength and strain at break were decreased regardless of methods. With the SUS method, the flexural strengths of H1C1 and H2C1 composites were decreased 9.3% and 27.4%, respectively, from 182 MPa of C1 sample. This reduction in strength was mainly due to a strong HA agglomeration, causing the growth of microcracks from applied external forces. However, the flexural strengths of H1C1 and H2C1 composites prepared by MF process were only decreased 3.8% and 7.0%, respectively, implying that the MF process was able to well pulverize HA aggregates and enhance HA dispersibility in the polymer matrix. Thus, the HA reinforced composites prepared by the MF method showed much improved flexural strength compared to the ones prepared by the SUS method, particularly H2C1 sample, which improved 30%. Compared to the flexural strength values of cortical bone that ranged from 103–238 MPa [45], the flexural strength of H2C1 composite was found to match the middle range. This could be further increased with the addition of CF filler. The strain to failure value of H2C1 composite prepared by the MF method was closely matched to the value of cortical bone that ranged from 1–3% [46]. The flexural modulus of PEEK composites from both SUS and MF methods increased as HA content was increased. The lamellar microcrystals between the PEEK matrix and the HA nanoparticles increased the modulus of elasticity by reducing the elastic resistance [29].

Table 3 showed the compressive strength data of PEEK composites. Similar to the flexural properties in C1, C2, and C3 samples, the compressive strength and modulus were also increased with the addition of CF filler. However, there was no significant change in the flexural properties between the C1, C2, and C3 composites prepared by SUS or MF method. After the incorporation of the HA filler, the compressive strength was improved by both methods. Among various bioactive ceramics, HA, which has low porosity and dense structure, could increase compressive strength with increasing content [47]. The composites prepared by the MF method showed higher compressive strength than the ones prepared by the SUS method because of the enhanced pulverization and dispersibility of the HA nanofiller. The compressive strength of H2C1 sample made by the MF method found to be in the middle range of cortical bone (i.e., 106–215 MPa) [48] and could be further improved with the addition of CF filler.

### 3.3. Morphology of Composites

**Figure 3:**
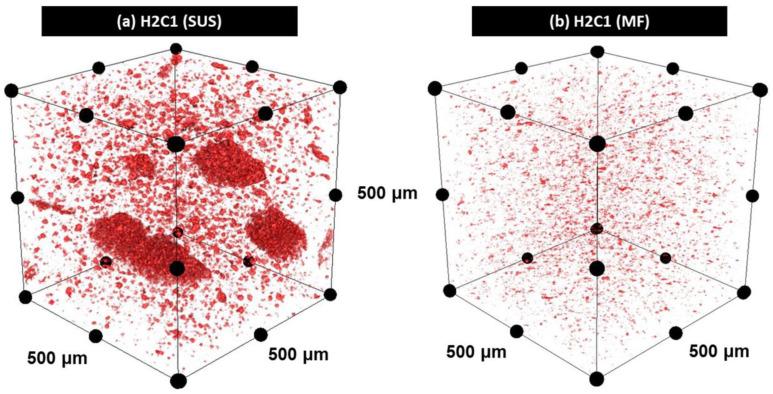
Three-dimensional X-ray micro-CT analysis on H2C1 composites made by (**a**) SUS and (**b**) MF.

Three-dimensional (3D) X-ray micro-CT analysis is a non-destructive investigation method used to precisely examine the internal structure of polymer composites [49,50]. Figure 3 showed the dispersion of the HA filler in H2C1 composite by using the 3D X-ray micro-CT. The observed dimensions of the composites were 0.5 × 0.5 × 0.5 mm^3^. The average size of the HA filler in H2C1 composite by the SUS method (Figure 3a) was ≤100 μm, indicating a strong aggregation of HA particles. Moreover, a number of the HA aggregates that are larger than 400 μm were observed. In contrast, the average size of the HA filler by the MF method was ≤20 μm, implying small aggregations since the original average size of HA in the technical specification was about 150 nm. HA was better dispersed by the MF method (Figure 3b) than by the SUS method due to enhanced shear, compressive, and frictional forces. It can be concluded that the strong mechanical forces during MF process could enhance the dispersibility of HA filler, thereby improving both flexural and compressive strength of the PEEK/HA/CF ternary composite.

**Figure 4:**
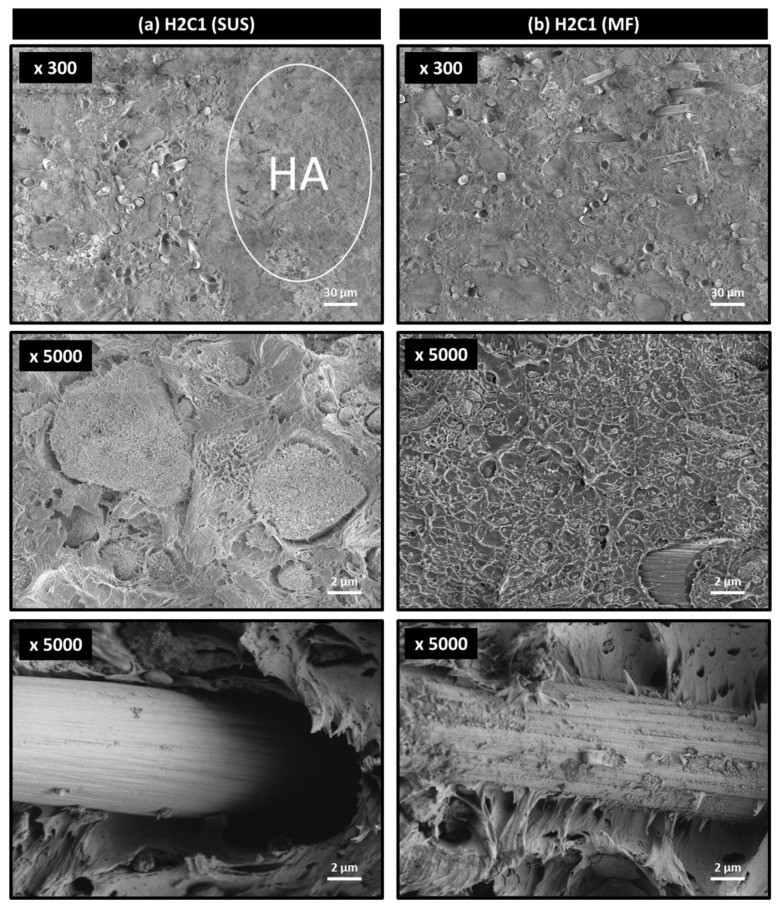
Fracture morphologies of H2C1 composites made by (**a**) SUS and (**b**) MF methods.

The fractured sections of H2C1 composite after flexural testing were observed to investigate the dispersibility of the HA filler. As shown in Figure 4, the fracture morphologies of both composites were changed from ductile to brittle failure due to HA and CF fillers. It can be seen that the main fracture mechanism occurred by aggregation of HA fillers. It was important to note that severely aggregated HA fillers were easily observed in the H2C1 composite made by the SUS method (Figure 4a), whereas the HA fillers in the H2C1 composite made by the MF method were dispersed better with a size of about 1 μm (Figure 4b). The poor interfacial interaction between HA and PEEK matrix was another reason for early failure of composites. In both H2C1 composites, the majority of the HA particles showed adhesive failure due to interfacial de-bonding with the PEEK matrix.

In the case of micro-sized CF filler, PEEK/HA/CF composite fabricated using HA covered PEEK and CF exhibited improved interfacial adhesion via the entanglement of interfacial HA particles, acting as a bridging material between PEEK and CF. The improved strength of the interfacial adhesion between PEEK matrix and CF fillers indicated the stresses applied to the composites were transferred from the PEEK matrix to the CF fillers, resulting in improved mechanical strengths of PEEK/HA/CF composite prepared by the MF method. These results demonstrated that MF method could improve flexural and compressive strengths of PEEK/HA/CF ternary composite by enhancing dispersion of the HA aggregates and interfacial adhesion between matrix and fillers.

### 3.4. Bioactivity

**Figure 5:**
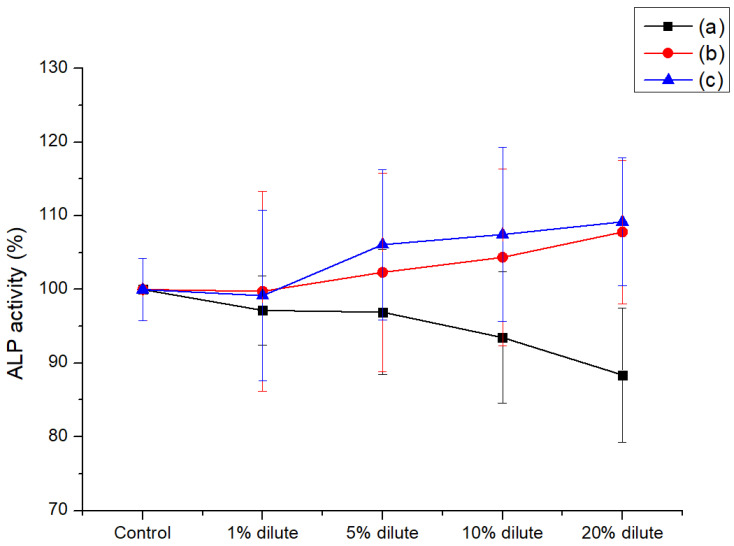
Cell activity results of PEEK composites via the ALP assay; (**a**) neat PEEK, (**b**) H2C1 (SUS), (**c**) H2C1 (MF).

Figure 5 showed the ALP activity behavior of HOB cells with various dilute concentrations (1, 5, 10, and 20%). Every composite showed high level of ALP activity above 97% at 1% dilute concentration. In particular, H2C1 composite (Figure 5b,c) showed a great increase of cell activity compared to the neat PEEK (Figure 5a). As the diluted concentration increase from 1 to 20%, the ALP activity behavior of H2C1 composite was increased above 100%, but the neat PEEK was decreased below 90%. The alkaline phosphatase activity in HOB cells depended on the formation of new bone under in vitro conditions. In addition, HA is a calcium phosphate material that has an osteo-compatibility, implying good bone formation properties [51,52]. Therefore, the ALP activity of H2C1 composite was higher than that of neat PEEK. Moreover, both H2C1 composites containing HA and CF of the same concentration, were retained a similar level of ALP activity behavior, although the degree of dispersion is different by the dispersion method.

**Figure 6:**
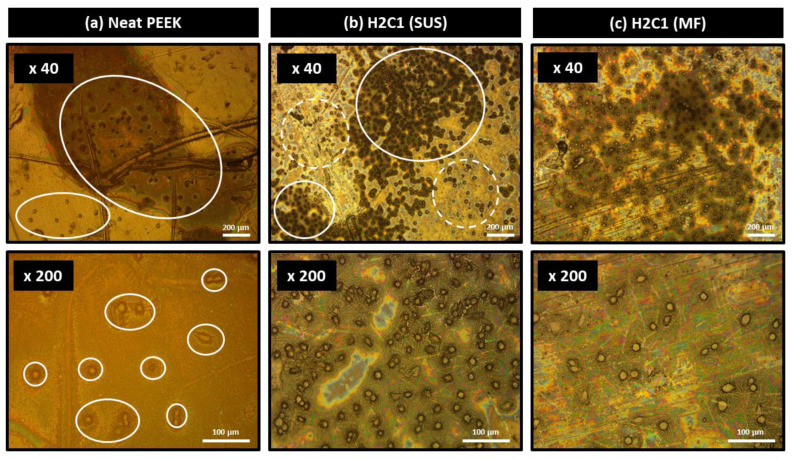
Optical microscope images of cell adhesion morphology on the substrates of PEEK composites; (**a**) neat PEEK, (**b**) H2C1 (SUS), (**c**) H2C1 (MF).

**Figure 7:**
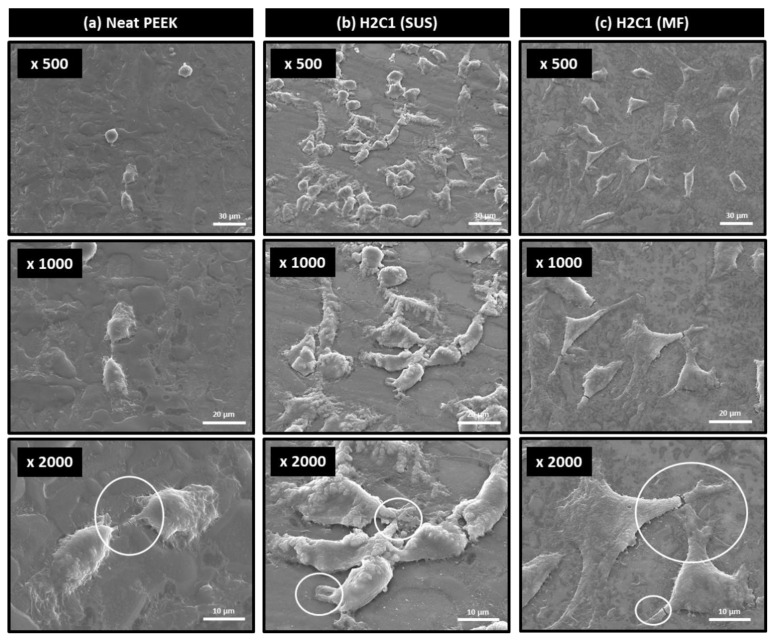
SEM images of cell morphologies on the PEEK substrates; (**a**) neat PEEK, (**b**) H2C1 (SUS), (**c**) H2C1 (MF).

For the application as bone cage materials, osteo-compatible HA should be well-dispersed on the surface of the composite. The HOB cell adhesion behavior of neat PEEK and H2C1 composites was determined by optical microscope and SEM. In Figure 6, the HOB cells were attached well on the surfaces of H2C1 composites (Figure 6b,c) compared to the neat PEEK (Figure 6a). Furthermore, the HOB cells on the surface of H2C1 composite made by the MF method were more finely dispersed than SUS method. These results indicated that the MF method is more suitable for dispersing HA particles than the SUS method. Figure 7 showed the morphologies of HOB cells that were attached to the surface of PEEK composites by SEM. In the stage of cell adhesion, cells form filopodia and lamellipodia on the surface. Filopodia and lamellipodia are important to determine the attached cell migration [53,54]. The cells on the neat PEEK (Figure 7a) were small and round, whereas the cells on the H2C1 composites were well spread out (Figure 7b,c). But, the HOB cells on the H2C1 composite made by SUS were aggregated. The cells attached to the H2C1 composites exhibited pseudopodia extension with filopodia and lamellipodia. Based on these results, the MF method was expected to be better method for bone cage application than the SUS method due to the enhanced dispersibility of the HA particles.

## 4. Conclusions

Comparison in mechanical properties of PEEK/HA/CF ternary composite fabricated through SUS and MF methods revealed that composite from the latter method had better properties. Higher flexural and compressive strengths and elongation at break were achieved through MF technique as compared to dispersion in ethanol. Improvements in dispersibility of the HA particles and interfacial adhesion between components were seen through 3D X-ray micro-CT and SEM micrographs, indicating a good blending of fillers with the polymer matrix. The H2C1 composite made by the MF method was found to match the middle range of cortical bone in both flexural and compressive strength. Moreover, in vitro cell compatibility and cell attachment tests exhibited improvement in bioactivity and osteo-compatibility due to enhanced dispersion of the HA particles by the MF process. The results demonstrated MF method as a better fabrication process for producing PEEK/HA/CF ternary composite compared to ethanol mixing, with the advantages of solvent-free, better processability, and cost-effectiveness.

## Figures and Tables

**Table 1:** Properties of materials.

	PEEK	HA	CF
Product name	Victrex 450PF	Nano HAP04	PX 35
Diameter/Length	50 μm	20 nm/150 nm	7.2 μm/150 μm
Tensile strength	98 MPa	−	4137 MPa
Tensile modulus	4 GPa	−	242 GPa
Flexural strength	165 MPa	−	−
Flexural modulus	3.8 GPa	−	−
Compressive strength	125 MPa	−	−
Density	1.3 g/cm^3^	−	1.81 g/cm^3^

**Table 2:** Formulation ratios of the PEEK composites.

Sample Code	PEEK Content (wt%)	HA Content (wt%)	CF Content (wt%)
PEEK	100	0	0
C1	90	0	0
C2	80	0	20
C3	70	0	30
H1C1	80	10	10
H1C2	70	10	20
H1C3	60	10	30
H2C1	70	20	10
H2C2	60	20	20

**Table 3:** Mechanical properties of PEEK composites.

	Flexural Modulus (GPa)		Flexural Strength (MPa)		Flexural Strain at Break(%)		Compressive Modulus (GPa)		Compressive Strength (MPa)	
	SUS	MF	SUS	MF	SUS	MF	SUS	MF	SUS	MF
PEEK	3.7 ± 0.2		155 ± 3		−		2.7 ± 0.1		120 ± 2	
C1	5.6 ± 0.1	5.7 ± 0.3	182 ± 3	185 ± 3	5.5 ± 0.3	5.4 ± 0.2	3.5 ± 0.1	3.6 ± 0.1	134 ± 1	135 ± 3
C2	8.7 ± 0.2	8.9 ± 0.3	219 ± 2	221 ± 2	3.8 ± 0.3	3.9 ± 0.1	4.0 ± 0.1	3.9 ± 0.1	151 ± 3	153 ± 4
C3	13.5 ± 0.4	13.5 ± 0.4	252 ± 5	254 ± 4	2.5 ± 0.1	2.4 ± 0.1	4.5 ± 0.1	4.4 ± 0.1	168 ± 2	170 ± 3
H1C1	7.7 ± 0.1	7.3 ± 0.1	165 ± 4	178 ± 1	2.4 ± 0.1	4.0 ± 0.1	2.3 ± 0.1	2.4 ± 0.1	139 ± 2	149 ± 2
H1C2	11.0 ± 0.2	10.9 ± 0.2	198 ± 3	215 ± 1	2.1 ± 0.1	3.2 ± 0.1	4.1 ± 0.2	2.6 ± 0.1	158 ± 2	170 ± 3
H1C3	18.2 ± 0.9	17.7 ± 0.3	228 ± 7	246 ± 2	1.5 ± 0.1	1.9 ± 0.1	4.6 ± 0.3	3.2 ± 0.2	178 ± 2	198 ± 3
H2C1	8.8 ± 0.1	8.9 ± 0.4	132 ± 3	172 ± 2	1.6 ± 0.1	2.4 ± 0.1	3.8 ± 0.1	3.2 ± 0.2	151 ± 5	167 ± 3
H2C2	13.3 ± 0.2	13.5 ± 0.1	163 ± 13	206 ± 3	1.4 ± 0.1	1.8 ± 0.1	4.5 ± 0.1	3.4 ± 0.1	170 ± 6	192 ± 3

## Data Availability

The data presented in this study are available on request from the corresponding author.
